# Bat skin microbiome and the association of ecoregion and bat species that impacted isolation of members with bioactivity against *Pseudogymnoascus destructans*

**DOI:** 10.1186/s42523-026-00591-4

**Published:** 2026-06-25

**Authors:** Paris S. Salazar-Hamm, María-José Romero-Jiménez, Nicole A. Caimi, Kevin R. Amses, Jennifer J. Marshall Hathaway, Debbie C. Buecher, Ernest W. Valdez, Lindsay K. Caesar, Andrea Porras-Alfaro, Diana E. Northup

**Affiliations:** 1 Department of Biology, New Mexico Institute of Mining and Technology, Socorro, New Mexico USA; 2 Department of Biology, University of New Mexico, Albuquerque, New Mexico USA; 3 Department of Biological Sciences, Western Illinois University, Macomb, Illinois USA; 4 Department of Botany and Plant Pathology, Oregon State University, Corvallis, Oregon USA; 5 Department of Microbiology, Perelman School of Medicine, University of Pennsylvania, Philadelphia, USA; 6 Buecher Biological Consulting, Tucson, Arizona USA; 7 U.S. Geological Survey, Fort Collins Science Center, Albuquerque, New Mexico USA; 8 Department of Chemistry and Biochemistry, James Madison University, Harrisonburg, Virginia USA; 9 Directorate for Biological Sciences, National Science Foundation, Alexandria, Virginia USA

**Keywords:** White-nose syndrome (WNS), White-nose disease (WND), *Actinomycetota*, Antifungal, *Streptomyces*, Microbiome

## Abstract

**Background:**

Microbiome constituents can serve as a primary defense against vertebrate pathogens. This may be crucial to the protection of North American bats who have been devastated over the last two decades by the fungal pathogen, *Pseudogymnoascus destructans (Pd)*, which causes white-nose syndrome (WNS). The objectives of this study were to isolate members of the external microbiome of bats post-hibernation in Arizona and New Mexico before the arrival of WNS, to identify those bacteria with bioactivity against *Pd*, and to determine the variables associated with antifungal strains.

**Results:**

In this study we isolated 2,936 bacteria from the fur and skin of 314 bats across 12 bat species at 6 sites across New Mexico and Arizona from 2013 to 2016. Selective media were used to promote the isolation of *Actinomycetota* (2,594 isolates, 88.4%), particularly species of *Streptomyces* (2,045 isolates, 69.7%) known for their bioactivity. Although *Actinomycetota* isolation was targeted, this collection includes representatives from *Pseudomonadota (Alpha-, Beta*-, and *Gamma-Proteobacteria*), *Bacillota*, and *Bacteroidota* phyla also common to the bat microbiome. A bi-layer challenge assay performed on 1,089 isolates identified 61 bat-associated bacteria with activity against Pd. These results were pooled with efforts by Hamm et al. [1] to determine what metrics impacted isolation of bacteria that inhibit *Pd* (*n* = 97). Ecoregion and bat species were determinant variables associated with *Pd* inhibition.

**Conclusions:**

This study represents, to our best knowledge, the largest culture collection of bacteria from bats’ skin and fur before the impact of WNS. We identified bacteria with antifungal activity against *Pd* and found significant associations of bioactive strains with bat species and ecoregions.

**Supplementary Information:**

The online version contains supplementary material available at https://doi.org/10.1186/s42523-026-00591-4.

## Background

In the last two decades there has been a heightened recognition of emerging infectious diseases, mounting concern for animal and human health, and acknowledgement of global biodiversity fragility [2–5]. One of the most notable catastrophic animal die-offs was caused by the fungus *Pseudogymnoascus destructans (Pd)*, the causal agent of white-nose syndrome (WNS). This wildlife disease has led to mass mortality and unprecedented population declines in many bat species across the U.S. and Canada [6–9]. *Pd* has the ability to invade bat skin causing ulcerations on the wings, uropatagium, ears, and muzzle [10, 11], which severely compromise vital regulatory functions, including thermoregulation, gas exchange, and water balance [12, 13]. In response, studies have aimed to understand the composition of the bat skin microbiome and its potential role in resistance and susceptibility of pathogens. In vivo treatment experiments on captive bats [14] have progressed to field trials for one probiotic bacterium *Pseudomonas fluorescens* [15]. While this has demonstrated promising results, it is limited to one bacterial strain augmented on one bat species (i.e. little brown bat, *Myotis lucifugus*), and researchers have yet to identify the bioactive compound(s). Additional studies of the bat microbiome may provide insights on natural resistance, other putative probiotic candidates, and synergistic activity of the microbial community.

In this study, we expanded greatly on our previous research [1], by building a robust culture collection of bacterial members of the bat microbiome pre-WNS (2013–2016) in New Mexico and Arizona. Low-level occurrences of *Pd* in New Mexico have been documented since 2018, but WNS was not reported in New Mexico until 2021 when two *Pd* detections were validated on cave walls in De Baca and Lincoln counties [16]. In 2023, WNS was confirmed in these same two locations when individuals of *Myotis thysanodes* (fringed bat) in Lincoln County and *Myotis velifer* (cave bat) in De Baca County were found dead from WNS [17]. In May of 2024, several *M. velifer* bats at Carlsbad Caverns National Park (CAVE) in Eddy County, New Mexico tested positive for *Pd* [18]. Recent surveillance in 2025 found 18 bats were *Pd* positive from CAVE including *M. velifer* bats as well as *M. thysanodes*, *Parastrellus hesperus* (western canyon bat), and *Tadarida brasiliensis* (Brazilian free-tailed bat) [19]. In Arizona, low-level occurrences of *Pd* were detected in four counties (Mohave, Coconino, Yavapi, and Cochise) in 2019 [20]. Between 2021 and 2024, surveillance efforts across Arizona found trace amounts of *Pd* on eight bats including species of *Eptesicus fuscus* (big brown bats), *Myotis auriculus* (southwestern bats), and *M. velifer* [20]. In June 2024, a single *M. velifer* bat in Fort Huachuca (Cochise County, Arizona) was confirmed with *Pd* and symptoms consistent with WNS including wing abnormalities [20]. Because *Pd* appears to be primarily transmitted by direct contact between bats and through contact with contaminated environmental surfaces [21], there is much concern over the imminent propagation of WNS to the southwestern United States (U.S.). Here, we isolated and identified 2,936 bat-associated bacteria from New Mexico and Arizona collected before the arrival of WNS. This provides essential baseline information on the bat bacterial microbiome before impact or alteration by invasive species such as *Pd*. A subset of 1,089 cultures were tested for antifungal capabilities against *Pd *in vitro and various metrics were examined for a positive correlation with antifungal activity. Superficial bacterial communities are in part shaped by the local environment [22–25], thus we will identify localities and bat species that have more bacteria with anti-*Pd* activity and may serve as protection against foreign invaders.

## Methods

### Study sites

**Fig. 1:**
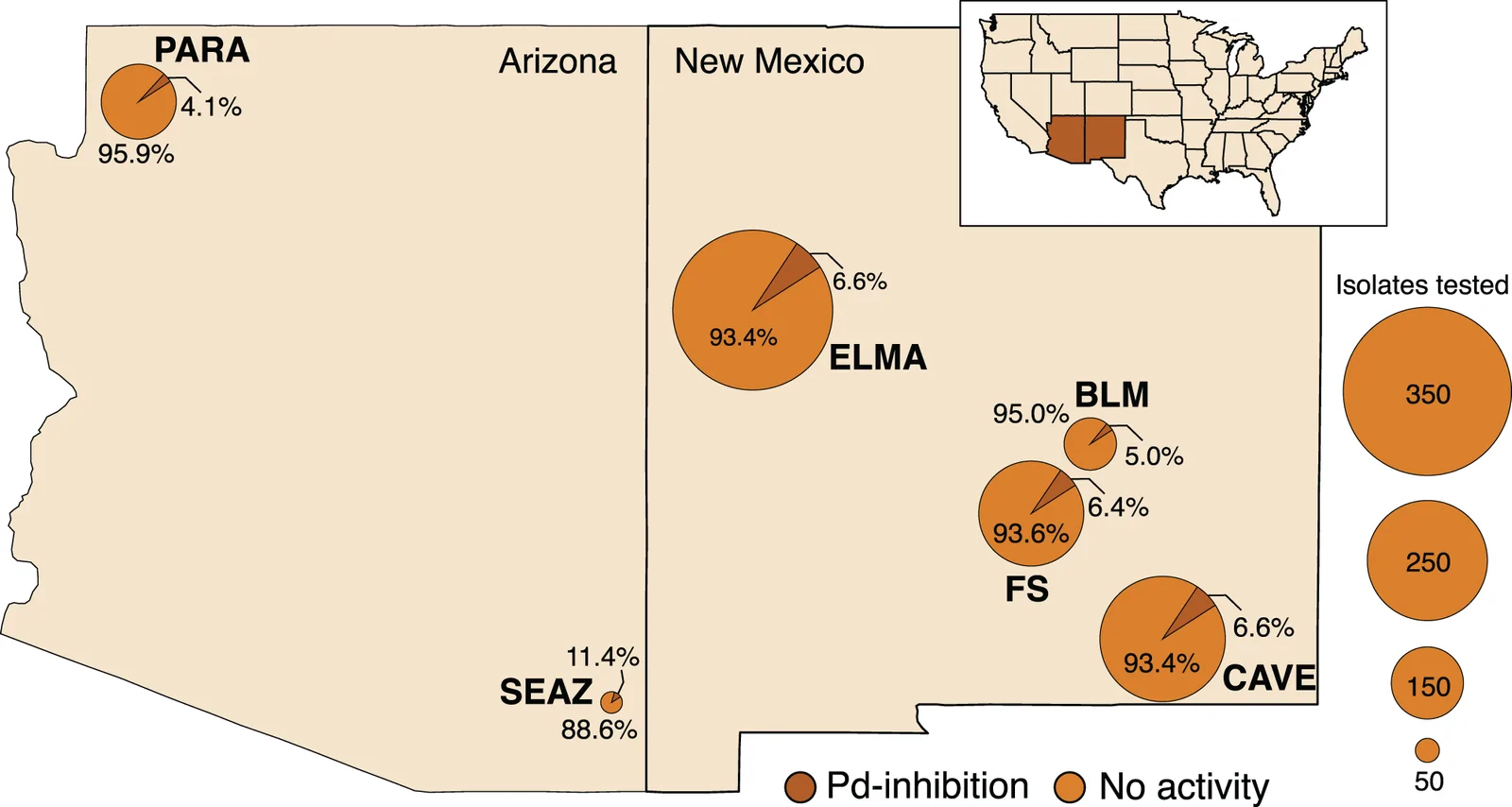
Sites in New Mexico and Arizona where bat-associated bacteria were obtained. Pie charts denote the percentage of isolates at these sites that inhibited *Pseudogymnoascus destructans* growth from the combined dataset of this study and Hamm et al. [1]. Pies are proportional to the number of isolates screened for bioactivity

Six study sites across Arizona and New Mexico were chosen to encompass the bat diversity and their associated microbial diversity in the southwestern U.S. (Fig. 1). Table 1 provides a comprehensive description of the study sites where bats were captured and swabbed for isolation of bacteria. Grand Canyon-Parashant National Monument (PARA) is the largest site in this study, situated in the northwestern corner of Arizona where seven surface localities across five ecoregions were sampled [26]. Southeast Arizona Group of National Park Sites (SEAZ), aptly named for its location in the southeastern corner of Arizona, had samples taken at both carbonate caves (two in Fort Bowie National Monument) and surface localities (three in Chiricahua National Monument) [27]. At El Malpais National Monument (ELMA), located in northwestern New Mexico west of Acoma Pueblo, we sampled ten lava caves [22, 28]. Additionally, bats were caught at three surface stock ponds located in El Malpais National Conservation Area, also grouped with ELMA. Fort Stanton-Snowy River Cave National Conservation Area (FS), found in southcentral New Mexico at the northern end of the Sacramento Mountains, contained two sampling locations (Fort Stanton carbonate cave and surface caught at Rio Bonito) [29, 30]. Two Bureau of Land Management (BLM) sites, gypsum Caves 45 and 55, located in southcentral New Mexico, were sampled [31, 32]. Carlsbad Caverns National Park (CAVE), our final site, is in southeastern New Mexico within the Guadalupe Mountains. Swabs were collected from bats roosting within Left Hand Tunnel, a carbonate cave, and at two surface localities near Rattlesnake Springs and Walnut Canyon Indian Ruins Pool [33, 34]. Five of these cave sites (CAVE, FS, BLM, PARA, and ELMA) had been surveyed previously [1]. However, in this study, we report the first survey of SEAZ and expand skin microbiome collections from the previously sampled locations. Sites were plotted using ggmap v3.0.0 [35].

**Table 1:** Site descriptions for six sampling sites across New Mexico and Arizona

	Grand Canyon-Parashant National Monument (PARA)	Southeast Arizona Group of National Park Sites (SEAZ)	El Malpais National Monument (ELMA)	Fort Stanton-Snowy River Cave National Conservation Area (FS)	Bureau of Land Management Sites (BLM)	Carlsbad Caverns National Park (CAVE)
State	Arizona	Arizona	New Mexico	New Mexico	New Mexico	New Mexico
Coordinates	36.5°N 113.5°W	32°N 109°W	35°N 108°W	33.5°N 105.5°W	34°N 105°W	32°N 104.5°W
Elevation	~375–2500 m.a.s.l	~1500–2000 m.a.s.l.	~2400 m.a.s.l	~1850 m.a.s.l.	~1550 m.a.s.l	~1000 m.a.s.l.
Ecoregions and Vegetation	Eastern Mojave Basin (sparsely populated desert scrubland); Eastern Mojave Low Ranges and Arid Footslopes (dominated by desert scrub creosote and sagebrush); Aubrey Montane Conifer Forest (conifers include Sonoran scrub and Gamble’s oak, pines, juniper, and aspen); Shivwits Woodland (juniper and pinon woods, ephedra shrubs, and various grama grasses); Arizona Strip Plateaus (transition zone between drier shrublands and forested mountain containing piñon, juniper, big sagebrush, and Mormon tea)	Apachian Valleys and Low Hills (valley plains, alluvial fans, and low hills with broadleaf tree species along creeks and sparse coniferous trees with cliffside grasses (e.g., cliff muhly, purple grama, and curly mesquite)	Lava Malpais (blue gramma, *Opuntia* and *Cholla* cacti, and herbaceous plants with sparse conifers)	Conifer Woodland and Savannah (piñon-juniper woodlands with various shrubs and grasses such as Stansbury cliffrose, Apache plume, fourwing saltbush, side oats grama, blue grama, and black grama)	Chihuahuan Basin and Playas (alluvial fans, internally drained basins, and river valleys that lend to desert shrubs and grasses such as creosote, tarbush, four wing saltbush, acacias, black grama, and alkali sacaton)	Chihuahuan Desert Grassland (succulents, arid grasses, and desert cacti); Chihuahuan Desert Slopes (scattered herbaceous wetlands with larger woody plants including ponderosa pine, chinkapin oak, alligator juniper, and bigtooth maple)
Surface localities	Lower Grand Wash Cliffs, Lower Last Chance, Mount Trumbull Guzzler, Pigeon Canyon, Tincannibits Tank, Waring Ranch, Whitmore Canyon	Rhyolite Creek, Shake Spring, Silver Spur	Cerro Rendeja, Cerro Rendeja II, Cerrito Comadre	Rio Bonito	N/A	Walnut Canyon Indian Ruins Pool, Rattlesnake Springs
Caves	N/A	Fort Bowie Ice Cave I and II	Cave 12, 18, 24, 25, 54, 61, 62, 110, 225, 261	Fort Stanton Cave	Cave 45 and 55	Left Hand Tunnel
Bat species^1^ (total bats)	ANPA (5), COTO (7), EPFU (3), LANO (2), MYCA (8), MYCI (5), MYTH (2), MYVO (2), PAHE (6), TABR (4)	COTO (3), EPFU (5), MYCA (2), MYEV (2), MYVE (2), MYTH (3)	COTO (44), EPFU (4), LANO (8), MYCI (14), MYEV (14), MYTH (2), MYVO (10), TABR (10)	COTO (19), EPFU (6), LANO (1), MYCI (1), MYTH (12), MYVO (1)	COTO (1), MYCA (1), MYCI (3), MYEV (10), MYTH (3), MYVE (47)	ANPA (7), COTO (2), MYTH (10), MYVE (19), PAHE (3), TABR (1)
Season sampled	Summer 2013, Spring 2014	Spring 2015	Spring 2013, Summer 2013, Spring 2014, Summer 2014, Spring 2015, Spring 2016	Fall 2013, Spring 2014, Fall 2015, Spring 2015	Fall 2013, Spring 2013, Spring 2014, Spring 2015, Spring 2016, Summer 2016	Summer 2014, Summer 2015

ANPA, *Antrozous pallidus*; COTO, *Corynorhinus townsendii*; EPFU, *Eptesicus fuscus*; LANO, *Lasionycteris noctivagans*; MYCA, *Myotis californicus*; MYCI, *Myotis ciliolabrum*; MYEV, *Myotis evotis*; MYTH, *Myotis thysanodes*; MYVE, *Myotis velifer*; MYVO, *Myotis volans*; PAHE, *Parastrellus hesperus*: TABR, *Tadarida brasiliensis*

### Bat sampling

Bats were caught using mist nets or hand plucked from cave walls post-hibernation [36], following approved protocols and collection permits from federal, state, and institutional authorities. Species, sex, and reproductive condition were determined by physical examination after swabbing [37]. During the respective sampling efforts, the wings, muzzle, ears, and uropatagium were visually assessed for any tissue damage (necrosis), lesions, scarring or skin mottling that could be attributed to potential *Pd* infections [10, 38], but all bats were determined to be WNS-free. We recognize that *Pd* is most active during the winter months when bats hibernate, but to swab and disturb the bats during this time would be unethical, and thus sampling performed in the spring, summer, and fall was justified. Bats were identified by Buecher or Valdez using standard morphological features [39]. Skin (wing and tail membranes) and the fur of the bats were swabbed with sterile nylon tipped swabs wetted with sterilized Ringer’s solution and then streaked on media, as described below. A total of 314 bats from Arizona and New Mexico were sampled and identified to 12 species (in order of abundance): Townsend’s big-eared bat (*Corynorhinus townsendii, n* = 76), cave bat (*Myotis velifer, n* = 68), fringed bat (*Myotis thysanodes, n* = 32), long-eared bat (*Myotis evotis, n* = 26), western small-footed bat (*Myotis ciliolabrum, n* = 23), big brown bat (*Eptesicus fuscus, n* = 18), Brazilian free-tailed bat (*Tadarida brasiliensis, n* = 15), long-legged bat (*Myotis volans, n* = 13), pallid bat (*Antrozous pallidus*, *n* = 12), silver-haired bat (*Lasionycteris noctivagans, n* = 11), California bat (*Myotis californicus, n* = 11), and western canyon bat (*Parastrellus hesperus, n* = 9) (Table 1). Abundance of bats was constrained by obtainability of populations roosting in the caves and bats captured by netting at aboveground localities.

### Isolation and identification of bacteria

*Actinomycetota*-specific media were used to isolate bacteria associated with the bat microbiome. Bats were swabbed in the field prior to the bat assessment, and culture plates were inoculated immediately onsite and stored in a cooler with ice for transport to the laboratory, where they were stored in a 10 °C incubator. Multiple media types were used to target bacteria including Actinomycete Isolation Agar (AIA, Difco^TM^, Sparks, MD, USA), Gellum Gum (GG, 7.0 g/L gellum gum, 7 mM calcium chloride), Humic Acid-Vitamin Agar (HVA [39]), or Glucose Yeast Extract Agar (GYEA). The original plates were sub-cultured to obtain single colony isolates on Reasoner’s 2A medium (R2A, Difco^TM^, Sparks, MD, USA). In efforts to target *Actinomycetota* and actively discourage the growth of Gram-negative bacteria and fungi, we supplemented the media with cycloheximide (50 mg/L), nalidixic acid (50 mg/L), trimethoprim (50 mg/L; Sigma-Aldrich, St. Louis, MO, USA), and a vitamin solution [40]. All cultures were grown at 20 °C to encourage growth of mesophilic bacteria but still within the upper limits of *Pd* growth [41]. Colonies from pure cultures were collected and transferred to 20% glycerol stocks for long-term storage at −80 °C at the University of New Mexico.

DNA from pure cultures was extracted using the MoBio UltraClean Microbial DNA Isolation kit (MoBio, Carlsbad, CA, USA) following the manufacturer’s protocol except substituting 1.5 minutes of bead-beating for the vortexing step. DNA was amplified with 8F (5′-AGAGTTTGATCCTGGCGCAG-3′) and 1492 R (5′-GGTTACCTTGTTACGACTT-3′) bacterial primers using polymerase chain reaction (PCR) [42]. Amplicons were purified with ExoSAP-IT® (Affymetrix, Santa Clara, CA, USA) and sequenced by Beckman-Coulter Genomics (Danvers, MA) or in-house with Big Dye 1.1, using primer 46F (5′-GCYTAAYACATGCAAGTCG-3′) for all sequences. From this study, 2,332 partial 16S rRNA sequences were deposited in GenBank under accession numbers MW568175–MW570505. In the analyses below we also included sequences previously published in Hamm et al. [1] (GenBank accessions: KX458184–KX458219 and KX928078–KX928646). All culture identification numbers and metadata are in Supplemental Table 1.

### Pd-inhibition assays

A bi-layer plate method previously described in [1] was employed to assess the in vitro ability of bacteria isolated from bats to inhibit *Pd*. A subset of 1,089 filamentous bacteria were selected for testing. We favored *Streptomyces* strains that are anticipated to have a higher potential for bioactivities. Each bacterium was streaked in the center of an R2A (Difco, Sparks, MD, USA) plate with a line 5 cm × 1 cm and allowed to incubate at 25 °C for 10 days. Next, Sabouraud dextrose agar (SDA; Oxoid, Basingstoke, Hampshire, England), cooled to 45 °C, was poured indirectly and slowly on top of the grown bacteria to not disturb the isolate. After the second layer had solidified, 100 µL of a *Pd* spore suspension (10^5^-10^7^ conidia/mL) was spread evenly over the SDA medium, and it was then incubated at 10 °C [41], for approximately three weeks. The *Pd* culture was obtained from a WNS-positive *Perimyotis subflavus* from Mesmore Cave, Indiana, USA isolated in 2010 and maintained at Western Illinois University (TW250 [43], Genbank accessions: KP902684 KP902702). For those with antifungal activity, the zone of inhibition was measured from the edge of the bacterial streak to the terminal growth of the fungus. Isolates were tested in triplicate and average zones of inhibition were ranked based on a scoring of low (<15 mm), medium (15–30 mm), or high (>30 mm) (Supplemental Table 2). Negative controls were made without any bacterial streak. Both negative controls and negative samples resulted in a complete lawn of *Pd* growth on the top layer and no zone of inhibition.

**Table 2:** Factors associated with members of the bat skin microbiome that can inhibit growth of *P. destructans*

	${\chi ^2}$	df	*p*-value
Sampling season	3.051	2	0.218
Ecoregion	29.43	10	0.001**
Site	5.773	5	0.329
Cave geology	4.581	3	0.205
Surface vs. cave	0.026	1	0.873
Bat species	23.27	11	0.016*
Bat sex	1.778	2	0.411
Media type	5.686	3	0.128

Variables were assessed using a Pearson’s Chi-squared (${\chi ^2}$) test from results of 1,540 bi-layer *Pd* antagonistic assays. Statistical significance was denoted * *p* < 0.05 and ** *p* < 0.01

The results from this study and those previously published by Hamm and colleagues [1] tested a total of 1,540 isolates from bats for antifungal activity against *Pd* using a bi-layer assay. We performed a t-test to investigate if there was a significant difference between these two datasets. Given that there was not, categorical data from these assays were pooled to create a heatmap of the 97 *Pd*-inhibitory bacterial isolates by ecoregion and bat species using ggplot2 v3.2.1 [44] in R v4.3.3 [45]. To test dependency between *Pd* inhibition and all variables from the pooled assays, we used a Pearson’s Chi-squared (${\chi ^2})$statistical analyses and post-hoc tests using the *chisq.test* and *chisq.posthoc.test* functions of STATS v4.5.1. Pairwise comparisons for each variable were corrected with the Benjamini-Hotchberg procedure (Supplemental Table 3). Biotic (bat species and bat sex), abiotic (sampling season, cave geology, surface versus cave capture, and culture media type), and geographical variables (site and ecoregion) were included. We utilized level III ecoregions, previously established, which denote ecosystems with the same type, quality, and quantity of environmental resources [46–48].

We further evaluated interactions among explanatory variables affecting the isolation of bacteria that inhibit *Pd* using Pearson’s Chi-squared test, followed by Cramér’s V using the *cramersV* function of the confintr package v1.0.2 to assess the strength of association. We then applied generalized linear modeling using a binomial distribution and the *glm* function of the STATS package v4.5.1 to determine whether these interactions were associated with *Pd* inhibition.

### Phylogenetic analyses

Both abundant and high in antifungal activity, *Streptomyces* isolates were further identified using 16S rRNA gene phylogenetic analysis. Eighty-four anti-*Pd Streptomyces* sequences generated in this study were compiled with published reference sequences [49] (Supplemental Table 4). *Micrococcus luteus* (GenBank accession NR_037113), *Nocardia salmonicida* (GenBank accession NR_041871), and *Catenulispora acidiphila* (GenBank accession NR_074457) were chosen as outgroups. Sequences were aligned with MAFFT v7.487 [50, 51] using automatically determined settings (i.e., *mafft -auto*). The resulting alignment was trimmed with trimal v1.4.rev22 [52] in -*automated1* mode. In addition, positions with <50% representation and sequences with <60% of representative positions were removed. The trimmed alignment consisted of 254 sequences covering 1,233 nucleotides. A maximum likelihood (ML) phylogeny was inferred under the transition model TIM3+F+R3, the best fitting model according to ModelFinder [53] in IQTree2 [54] with 100 nonparametric bootstrap replicates. The resulting best ML tree was visualized and annotated with ggtree and ggtreeExtra in R [55–57].

## Results

### Actinomycetota were readily cultured from bat skin microbiome

We leveraged 16S rRNA gene sequencing to identify 2,936 bacteria collected from the fur and skin of 314 bats within 12 bat species at six sites across New Mexico and Arizona (Fig. 1; Supplemental Table 1). Similar numbers of isolates were obtained from surface (1,475 isolates, 50.2%) and cave (1,461 isolates, 49.8%) caught bats. Cave roosting bats were sampled in three cave types: carbonate (607 isolates), lava (531 isolates), and gypsum (323 isolates) caves. Cultures were acquired using multiple media types with HVA (1,370 isolates) and AIA (1,018 isolates) providing the bulk of isolates, followed by GG (536 isolates) and GYEA (12 isolates). Although 12 bat species were sampled, the majority of isolates were obtained from *C. townsendii* (76 bats, 645 isolates, 22.0%) and *M. velifer* (68 bats, 532 isolates, 18.1%). This is due to the abundance of these bat species across southwestern U.S. caves. The remaining species provided less than 10% of the isolates each: *M. ciliolabrum* (23 bats, 287 isolates, 9.8%), *M. thysanodes* (32 bats, 273 isolates, 9.3%), *E. fuscus* (18 bats, 224 isolates, 7.6%), *A. pallidus* (12 bats, 189 isolates, 6.4%), *T. brasiliensis* (15 bats, 163 isolates, 5.6%), *M. evotis* (26 bats, 158 isolates, 5.4%), *M. volans* (13 bats, 146 isolates, 5.0%), *L. noctivagans* (11 bats, 122 isolates, 4.2%), *M. californicus* (11 bats, 104 isolates, 3.5%), and *P. hesperus* (9 bats, 93 isolates, 3.2%).

**Fig. 2:**
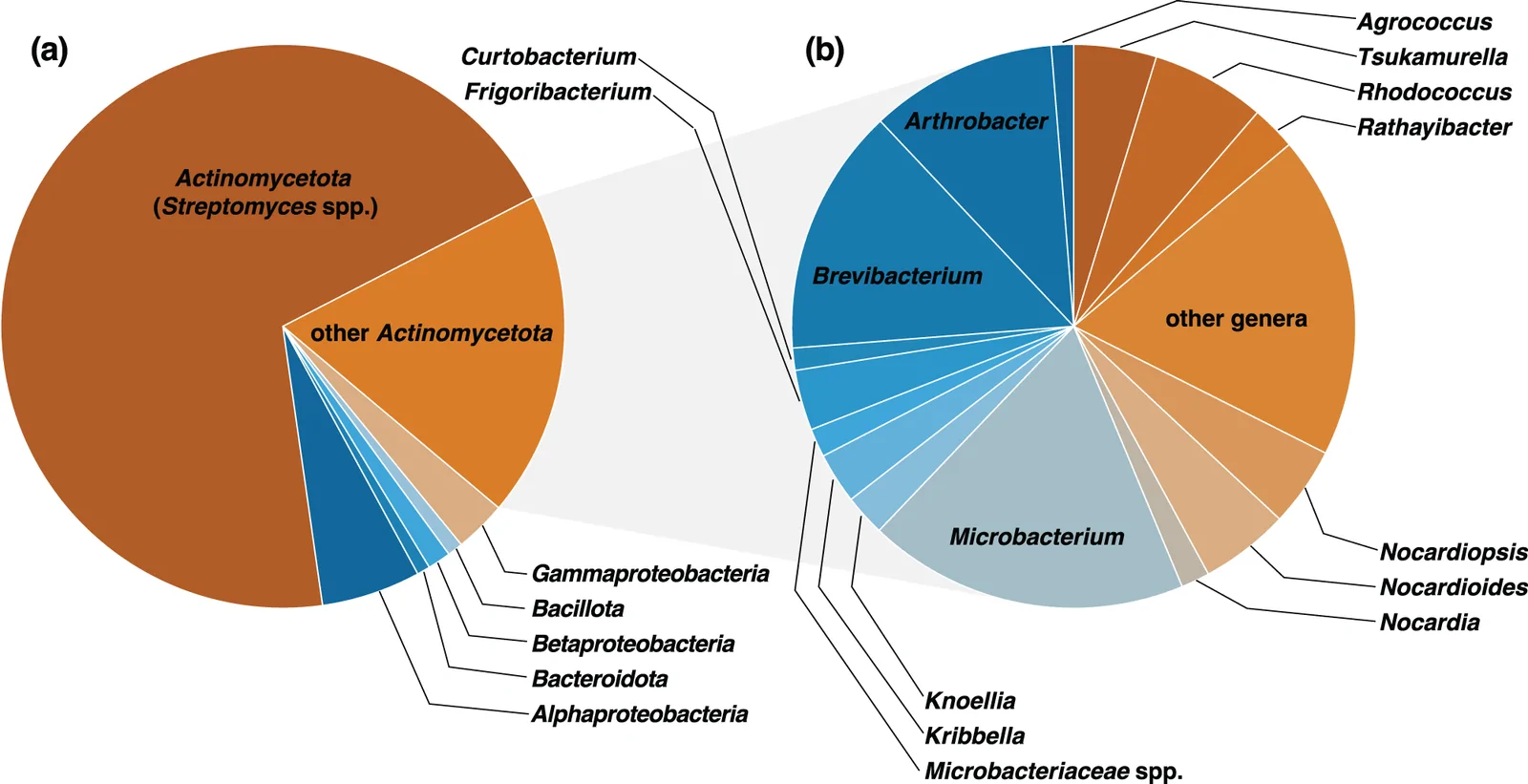
Taxonomic classification of 2,936 cultures from the bat skin microbiome. (**a**) Majority of the isolates fell within the phylum *Actinomycetota* with a large portion within the genus *Streptomyces*. Members were also within the phyla *Pseudomonadota* (*Alphaproteobacteria*, *Gammaproteobacteria*, and *Betaproteobacteria*), *Bacillota*, and *Bacteroidota*. (**b**) There was a great diversity of other taxa within the genus *Actinomycetota*

As anticipated, the cultures were dominated by members of *Actinomycetota* (previously *Actinobacteria*) (2,594 isolates, 88.4%), and the most abundant genus was *Streptomyces* (2,045 isolates, 69.7%) (Fig. 2). There were 549 other strains of *Actinomycetota* which included the genera *Microbacterium* (101 isolates), *Brevibacterium* (78 isolates), *Arthrobacter* (59 isolates), *Rhodococcus* (36 isolates), *Nocardioides* (28 isolates), and *Tsukamurella* (25 isolates), among others (Fig. 2B). Despite selectively targeting *Actinomycetota*, we isolated members of *Pseudomonadota* (previously *Proteobacteria*) (10%), mainly *Alphaproteobacteria* (167 isolates, 6%) followed by *Gammaproteobacteria* (87 isolates, 3%) and *Betaproteobacteria* (39 isolates, 1%) (Fig. 2A). Additionally, a small percentage of isolates were members of *Bacillota* (previously *Firmicutes*) (26 isolates, 1%) and *Bacteroidota* (previously *Bacteroidetes*) (22 isolates, 1%) (Fig. 2A).

### Bat-associated bacteria inhibit Pd

**Fig. 3:**
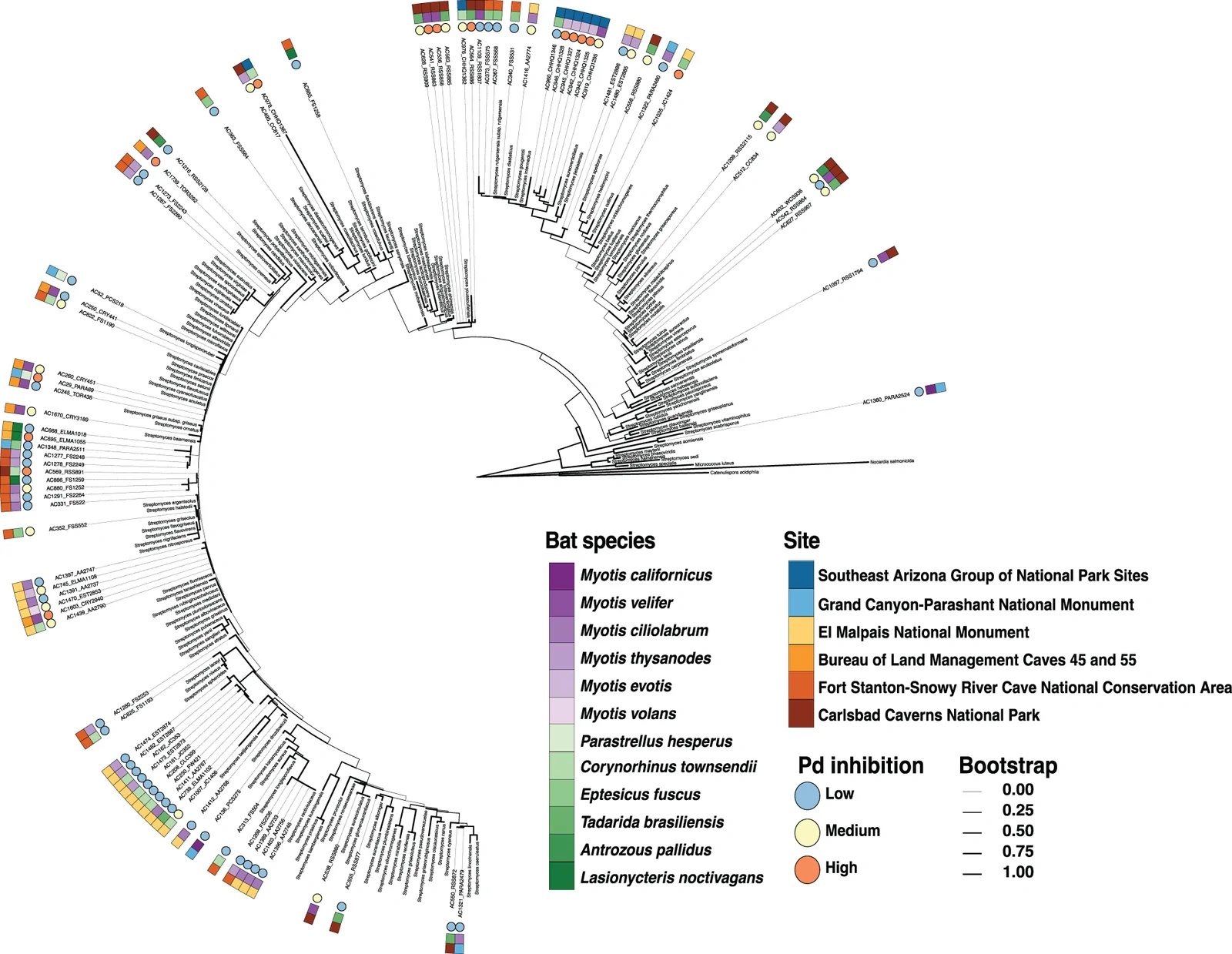
A maximum likelihood phylogenetic analysis of *Streptomyces* species that showed antifungal activity against *Pseudogymnoascus destructans* (*Pd*). Supplementary *Streptomyces* taxa were acquired from Labeda et al. [49] and *Micrococcus luteus*, *Nocardia salmonicida*, and *Catenulispora acidiphila* were chosen as outgroups. Bootstrap support based on 100 nonparametric replicates is indicated by edge thickness. Degree of inhibition is shown as colored circles. Additional site and bat species variables are shown in concentric heatmaps respectively. *Myotis* species were denoted in shades of purple while other bat genera are in green. Arizona sites are represented by the two shades of blue (Southeast Arizona group of National Park sites and Grand Canyon-Parashant National Monument) while New Mexico sites (El Malpais National Monument, Bureau of Land Management caves 45 and 55, Fort Stanton-Snowy River Cave National Conservation Area, and Carlsbad Caverns National Park) are yellow-orange hues

We assessed 1,089 cultures with a bi-layer assay for inhibition of *Pd*. This assay was most successful for testing of filamentous strains that could resist disturbance from a second layer of media and less so for mucoid morphologies. Sixty-one isolates (5.6%) had antifungal activity. Fifty-three (86.7%) of those bioactive strains were identified as species of *Streptomyces*. There were two bioactive *Brevibacterium* strains and one strain each of *Agrococcus*, *Arthrobacter*, *Methylobacterium*, *Rhizobium*, *Rhodococcus*, and *Streptosporangium* (Supplemental Table 2). Ten isolates had a high inhibition (>30 mm), 14 medium inhibition (15–30 mm), and 37 low inhibition (<15 mm) (Supplemental Table 2). Five (50%) of the high *Pd*-inhibitors were isolated from surface caught bats at SEAZ Rhyolite Creek, and four of these were from a single *M. evotis* (AC942_CHHQ1324, AC943_CHHQ1325, AC945_CHHQ1327, AC946_CHHQ1328). Our phylogenetic analysis revealed these *Streptomyces* strains were also closely related to a medium *Pd*-inhibitor isolate from a *C. townsendii* (AC919_CHHQ1295) at the same location (Fig. 3). In fact, we isolated a total of nine *Pd*-inhibitors from bats caught at SEAZ which had the highest *Pd*-inhibitor recovery rate of any site at 11.4%, over twice the average (Fig. 1). Eight of these *Pd*-inhibitors from SEAZ were identified as species of *Streptomyces* and the other was a species of *Brevibacterium*. The remaining high *Pd*-inhibitors were from BLM (*n* = 3) and ELMA sites (*n* = 2). One isolate, AC1638_TOR2983, a *Brevibacterium* sp. from a *M. velifer* in a BLM cave, was found to completely inhibit the growth of *Pd*.

### Ecoregion and bat species impact isolation of bacteria that inhibit Pd

For a more robust analysis of relevant variables impacting isolation of bacteria that inhibit *Pd*, we combined the results from this study with those from Hamm and colleagues which utilized the same bat sampling methods and in vitro bioactivity testing [1]. Using a t-test, we compared the performance of this assay from both datasets and found no differences (*p* = 0.730). Our combined dataset was 1,540 bat-associated isolates tested for anti-*Pd* activity with 97 (6.3%) demonstrating inhibition of the fungus (Supplemental Table 2). Seventeen had a high level of inhibition (>30 mm), 25 had medium inhibition (15–30 mm), and 55 had low inhibition (<15 mm). Of those that had a high level of inhibition, 14 were within the genus *Streptomyces* and one strain each of *Brevibacterium*, *Nocardiopsis*, and *Rhodococcus.* Four of these isolates, all species of *Streptomyces*, completely prevented the growth of *Pd* (AC29_PARA69, AC564_RSS886, AC569_RSS891, and AC1638_TOR2983). Two were isolated in CAVE, one in PARA, and one in BLM, and they were all from different bats species (*P. hesperus*, *T. brasiliensis*, *C. townsendii,* and *M. velifer*) (Supplemental Table 2). Similarly to above, *Streptomyces* was the most abundant genus identified with anti-*Pd* activity in the combined dataset (*n* = 85, 87.6%). This is in part due to the high number of *Streptomyces* isolates tested (1,095 tested, 7.8% anti-*Pd*). Additionally, *Pd*-antagonistic activity was documented for members of *Agrococcus*, *Arthrobacter*, *Brevibacterium, Luteipulveratus*, *Methylobacterium*, *Nocardiopsis*, *Rhizobium*, *Rhodococcus*, *Streptosporangium* (Supplemental Table 2). Other taxa had notable antifungal recovery rates even though there were a relatively low number tested including *Streptosporangium* (4 tested, 50% anti-*Pd*), *Agrococcus* (5 tested, 20% anti-*Pd*), and *Rhodococcus* (20 tested, 10% anti-*Pd*).

**Fig. 4:**
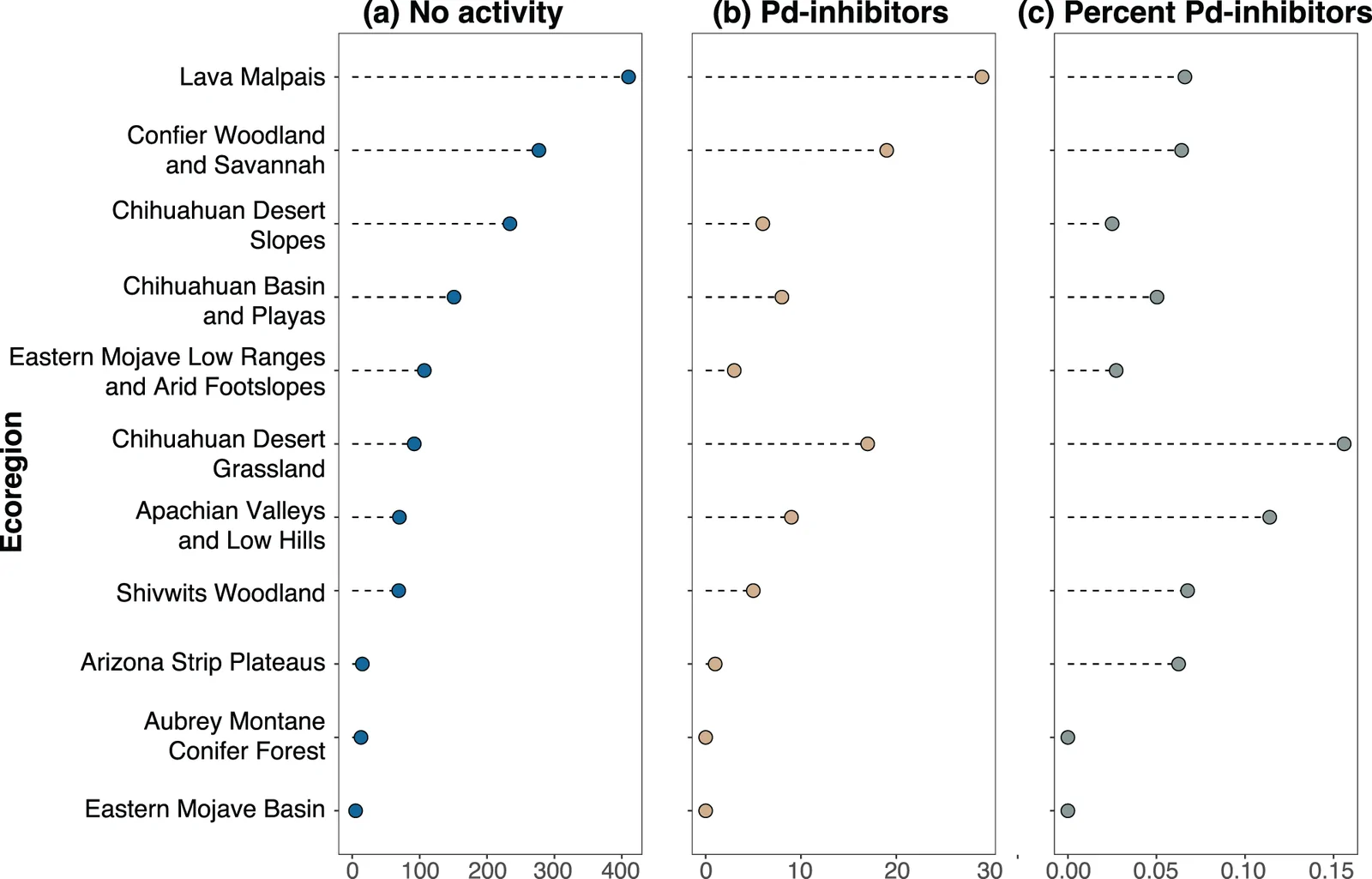
Isolates that were tested for bioactivity against *Pseudogymnoascus destructans* (*Pd*) categorized by ecoregion. Panels depict the (**a**) absolute value of those tested with no bioactivity (*n* = 1,443), (**b**) absolute value of those tested with anti-*Pd* activity (*n* = 97), and (**c**) percentage of isolates with anti-*Pd* activity

We found ecoregion (${\chi ^2}$ test, *p* = 0.001) was the strongest variable impacting isolation of bacteria that inhibit *Pd* (Table 2, Supplementary Table 4). Because of sampling availability, however, testing was uneven across the eleven ecoregions (Fig. 4A). All ecoregions had > 70 isolates tested except for Arizona Strip Plateaus (*n* = 16), Aubrey Montane Conifer Forest (*n* = 13), and Eastern Mojave Basin (*n* = 5) (Fig. 4A). The greatest number of *Pd*-inhibitors were recovered from Lava Malpais (29 *Pd*-inhibitors, 29.9%), Conifer Woodland and Savannah (19 *Pd*-inhibitors, 19.5%), and Chihuahua Desert Grasslands (17 *Pd*-inhibitors, 17.5%) ecoregions (Fig. 4B). Indeed, Lava Malpais had the most isolates tested (439 tested) followed by Conifer Woodland Savannah (296 tested). We then accounted for the number of isolates tested from each ecoregion and found the recovery rates were the highest in Chihuahuan Desert Grassland (15.6% *Pd*-inhibitors, 17 isolates out of 109) and Apachian Valleys and Low Hills (11.4% *Pd*-inhibitors, 9 isolates out of 79) (Fig. 4C). There were no anti-*Pd* isolates found in Eastern Mojave Basin and Aubrey Montane Conifer Forest ecoregions which was unsurprising since very few isolates were tested (Fig. 4A). Of those ecoregions with anti-*Pd* isolates, the lowest recovery rates were found in Eastern Mojave Low Ranges and Arid Footslopes (2.7%, 3 out of 110 isolates) and Chihuahuan Desert Slopes (2.5%, 6 out of 240 isolates) (Fig. 4C).

**Fig. 5:**
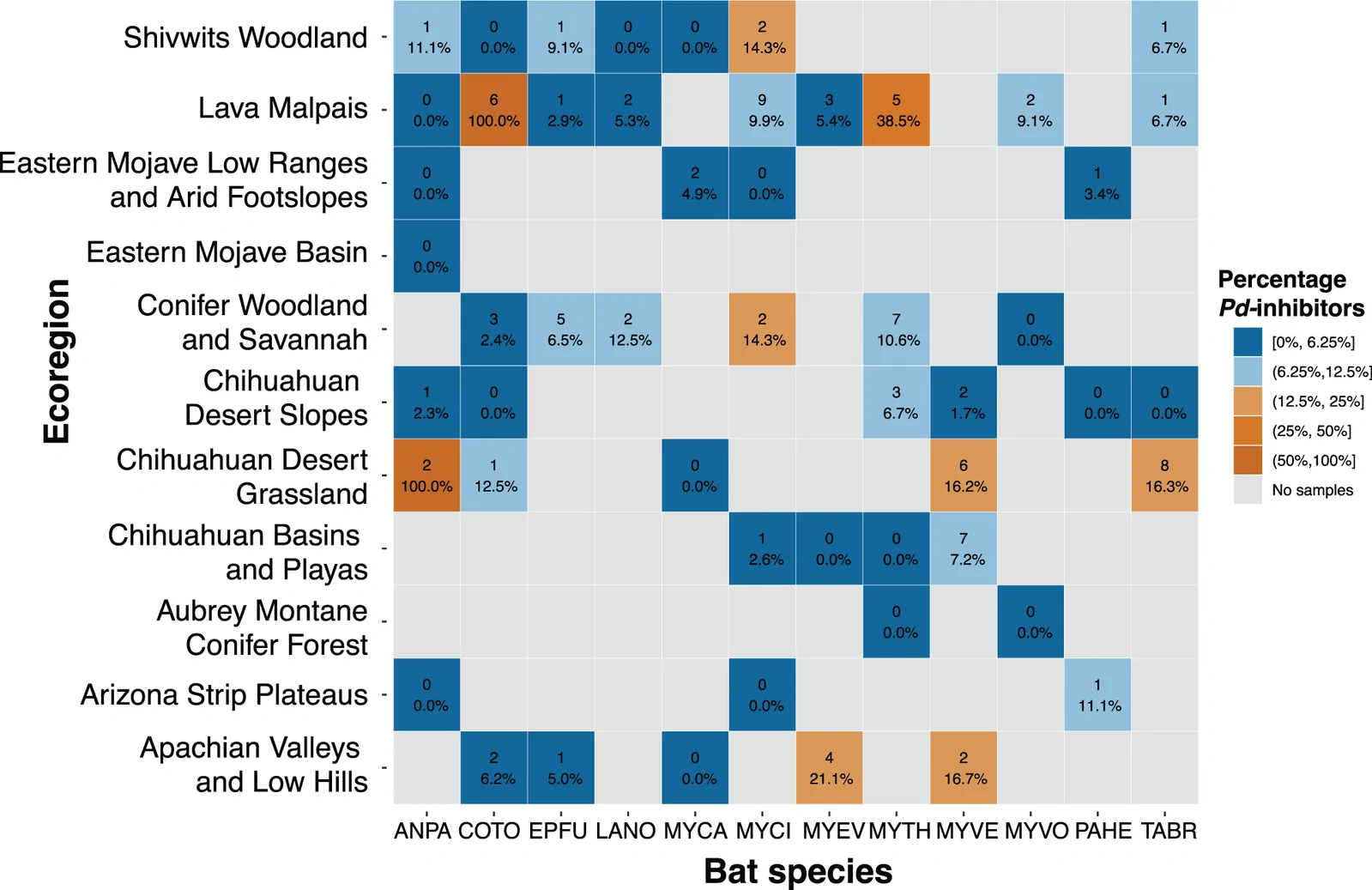
Intensity of *P. destructans* (*Pd*) antagonistic isolates for bat species within each ecoregion. When analyzed with a general linear model, we confirmed bat species (*p* = 0.001) and ecoregions (*p* = 0.002) were independently associated with *Pd* inhibition while their interaction was not (*p* = 0.235). Individual squares contain the number of *Pd*-inhibitors (top) and the percentage of *Pd*-inhibitors (bottom) at that ecoregion for a bat species. The color palette is log2 scaled and signifies low (dark blue) to high (burnt orange) percentage of *Pd*-inhibitors. Grey boxes indicate no isolates were obtained from a bat species within a certain ecoregion. Bat species sampled included: ANPA, *Antrozous pallidus*; COTO, *Corynorhinus townsendii*; EPFU, *Eptesicus fuscus*; LANO, *Lasionycteris noctivagans*; MYCA, *Myotis californicus*; MYCI, *Myotis ciliolabrum*; MYEV, *Myotis evotis*; MYTH, *Myotis thysanodes*; MYVE, *Myotis velifer*; MYVO, *Myotis volans*; PAHE, *Parastrellus hesperus*: TABR, *Tadarida brasiliensis*

The only additional variable impacting isolation of bacteria that inhibit *Pd* was bat species (${\chi ^2}$ test, *p* = 0.016) (Table 2, Supplementary Table 3). At least one isolate from every bat species examined (*n* = 12) had antifungal activity against *Pd* (Fig. 5). The highest number of anti-*Pd* isolates were collected from *M. velifer* (*n* = 17) followed by *M. thysanodes* (*n* = 15), *M. ciliolabrum* (*n* = 14), *C. townsendii* (*n* = 12), *T. brasiliensis* (*n* = 10), *E. fuscus* (*n* = 8), *M. evotis* (*n* = 7), *A. pallidus* and *L. noctivagans* (*n* = 4 each), and *P. hesperus*, *M. volans*, and *M. californicus* (*n* = 2 each). When accounting for number of isolates tested from each bat species, *T. brasiliensis* had the highest recovery rate of anti-*Pd* isolates (10.8%) with a critical proportion of these bacteria isolated from Chihuahuan Desert Grassland (8 *Pd*-inhibitors, 16.3% anti-*Pd*) (Fig. 5). *Myotis thysanodes* followed with an anti-*Pd* isolation rate of 10.3%, predominantly from Lava Malpais (5 *Pd*-inhibitors, 38.5%) and Conifer Woodland and Savannah (7 *Pd*-inhibitors, 10.6%) ecoregions (Fig. 5). Third and fourth highest recovery rates were *M. ciliolabrum* (8.4% anti-*Pd*) and *M. evotis* (7.5% anti-*Pd*). In Apachian Valleys and Low Hills, which had the second highest anti-*Pd* recovery rate of any ecoregion, *M. evotis* (4 *Pd*-inhibitors, 21.1%) and *M. velifer* (2 *Pd*-inhibitors, 16.7%) provided most of the isolates (Fig. 5). Four bat species, *L. noctivagans* (6.7% anti-*Pd*), *M. velifer* (6.5% anti-*Pd*), *C. townsendii* (6.3% anti-*Pd*), and *E. fuscus* (5.6% anti-*Pd*), fell within 1% of the average recovery rate (6.3%). The lowest anti-*Pd* isolation rates were found in *A. pallidus* (1.4% anti-*Pd*, 4 out of 294 isolates), *M. californicus* (2.9% anti-*Pd*, 2 out of 68 isolates), *P. hesperus* (3.4% anti-*Pd*, 2 out of 58 isolates), and *M. volans* (4.5% anti-*Pd*, 2 out of 44 isolates).

We further tested the interactions between bat species and ecoregions as explanatory variables, and we found that they were correlated (${\chi ^2}$ test, *p* = 2.2 × 10^−16^). A Cramér’s V test found that the association was 0.413 on a scale from 0 not associated to 1 perfectly associated. When analyzed with a general linear model, we confirmed bat species (*p* = 0.001) and ecoregions (*p* = 0.002) were independently associated with *Pd* inhibition while their interaction was not (*p* = 0.235).

Despite other variables explored for trends for isolating anti-*Pd* isolates, we determined no statistical significance for surface versus cave isolates (${\chi ^2}$ test, *p* = 0.873), bat sex (${\chi ^2}$ test, *p* = 0.411), sampling season (${\chi ^2}$ test, *p* = 0.218), cave geology (${\chi ^2}$ test, *p* = 0.205), or isolation media type (${\chi ^2}$ test, *p* = 0.128) (Table 2).

### Bat-associated Streptomyces strains exhibit genetic and bioactivity diversity

We included all *Streptomyces* with anti-*Pd* activity in a 16S rRNA gene phylogenetic analysis (Fig. 3). Several of the strains clustered together in genetically related clades. Some had differing antifungal strengths such as the *S. buecherae* clade (AC563_RSS885, AC536_RSS858, AC541_RSS863, and AC628_RSS909) of which two had medium inhibition and two had high inhibition (Fig. 3). Other clades such as the relatives of *S. rectiviolaceaus* (AC1268_FS2236, AC1389_AA2733, AC1403_AA2756, and AC1396_AA2746) showed consistent low-level *Pd* inhibition. Some clades of closely related isolates were obtained from the same site (i.e., ELMA, CAVE, or SEAZ specific clades) (Fig. 3). For example, seven closely related low-level inhibitors (AC1474_EST2874, AC1482_EST2887, AC162_JC353, AC1473_EST2873, AC161_JC352, AC208_CLC399, AC230_FW421) were specific to ELMA, but were sampled from five different caves and two bat species (*C*. *townsendii* and *M*. *thysanodes*). However, there were many other clades that contained isolates from multiple New Mexico and Arizona sites such as AC550_RSS872 isolated from *T. brasiliensis* in CAVE and AC1321_PARA2479 isolated from *M. ciliolabrum* in PARA (Figs. 1, 3). In the previous example too, closely related strains were isolated from multiple bat species. Altogether this suggests that anti-*Pd* strains may be shared across bat species and sites.

## Discussion

Here we describe, to our best knowledge, the largest collection of bacteria isolated from the bat skin microbiome. Although dominated by *Actinomycetota*, members of *Pseudomonadota* (*Gammaproteobacteria*, *Alphaproteobacteria*, and *Betaproteobacteria*), *Bacillota*, and *Bacteroidota* were also isolated (Fig. 2). These bacterial groups have been previously documented in bat microbiomes [23, 25, 58–60]. Although new technologies offer avenues to characterize microbiomes without the laborious isolation [61], physical cultures, like those in this study, are essential to describing novel bio- and chemo-diversity.

Although now thousands of microbial strains from bats have been tested against *Pd* [1, 62–67], to our knowledge, only two studies have investigated the chemical constituents responsible for this antifungal activity [64, 68]. Li and colleagues identified three volatile and one non-volatile constituent of antifungal *Pseudomonas* strains isolated from *Rhinolophus ferrumequinum* (greater horseshoe bat) and *Myotis petax* (eastern water bat) in China that had moderate in vitro activity against *Pd* [64]. More recently, *Streptomyces buecherae* (AC541_RSS863), isolated from a *M. velifer* at CAVE, was found to produce the polyether antimicrobial nigericin and several chemical derivatives thereof that significantly inhibit the growth of *Pd* in a disc diffusion assay at concentration ≥ 6.25 μg/disc [68]. Genomic analysis of *S. buecherae* strains (AC541_RSS863, AC563_RSS885, and AC536_RSS858) isolated from *M. velifer* and *T. brasiliensis* reveal between 31 and 35 biosynthetic gene clusters capable of producing diverse natural products, at least 20 of which likely encode uncharacterized natural product molecules [69]. This is consistent with other investigations of bacteria isolated from bats in the southwestern U.S. that confirm great genomic and biosynthetic novelty [70–72]. These findings reveal the untapped potential of bat-associated bacteria as a source of bioactive natural products.

Our culture-based screening of 1,089 bat-associated bacteria identified members of the bat skin microbiome with antifungal activity against *Pd*. Our recovery rate of bioactive cultures is consistent with other efforts that tested bat microbiota against *Pd* despite using alternate techniques [1, 62, 63]. We identified strains of several genera with previously reported bioactivity against *Pd*: *Arthrobacter* [63, 65], *Rhizobium* [63], *Rhodococcus* [1, 62, 63, 73], *Streptomyces* [1, 62, 63, 65], and *Streptosporangium* [1]. We additionally report newly detected *Pd*-inhibition by bat-associated strains of *Agrococcus*, *Brevibacterium*, and *Methylobacterium*. It is quite possible that many of these isolates have multiple bioactivities, and other members of these taxa report antibacterial, anti-inflammatory, anticancer, and antioxidant properties [74–77]. We pooled the bioactivity results from this study and Hamm et al. [1] for a combined dataset of 1,540 isolates to explore factors associated with the isolation of bacteria from bats that can inhibit *Pd.* Unlike similar efforts that screened bats in western Canada [62], we found that both geographic patterns (i.e., ecoregion) and host demographics (i.e., bat species) impacted isolation rate of anti-*Pd* bat-associated bacteria in the southwestern U.S. (Table 2). Interestingly, site did not have a significant influence, but ecoregion, which provides finer resolution based on similar landscapes and land usage, was significantly associated with isolation rates. Specifically, Chihuahuan Desert Grassland and Apachian Valleys and Low Hills ecoregions had the highest isolation rates of anti-*Pd* bacteria (Fig. 4C). Chihuahuan Desert Grassland was one of two ecoregions at CAVE, an important site where a renowned *T. brasiliensis* maternal colony roosts [78]. Currently, there are no known bat deaths in CAVE from WNS; however, first *M. velifer* bat tested *Pd* positive in 2024 [18], and in 2025, 18 bats tested *Pd* positive now including *M. thysanodes*, *M. velifer*, *P. hesperus*, and *T. brasiliensis* [19]. Interestingly, the two Arizona sites had the highest (SEAZ) and lowest (PARA) anti-*Pd* isolation rates (Fig. 1). All SEAZ isolates were in Apachian Valleys and Low Hills ecoregion, while PARA was divided into five ecoregions (Eastern Mojave Basin, Eastern Mojave Low Ranges and Arid Footslopes, Aubrey Montane Conifer Forest, Shivwits Woodland, and Arizona Strip Plateaus) (Table 1). However, all bats caught at PARA sites were from surface netting sites. Although *Pd* positivity was reported from in SEAZ and PARA since 2019 (about 2–4 bat cases annually) [20], it is still early in the establishment of WNS in both Arizona and New Mexico, and the disease has not yet hit with the intensity that affected the eastern U.S.

Several phylogenetically related *Pd*-antagonistic *Streptomyces* sequences were detected across multiple sites and bats; however, there were also clades limited to one bat species at one cave (Fig. 3). Bat skin microbiome composition has been shown to be strongly influenced by the geography, host species, and host behavior [23, 24, 58, 79]. Despite some New Mexico and Arizona sites located up to 1,500 km apart, some bats species execute seasonal movements, such as *M. lucifugus* (up to 650 km [80]) and *T. brasiliensis* (up to 1,500 km [81]), which could occasionally facilitate microbial dispersal of great distances. Roost switching [82] and colonial behavior [83] could account for dispersion and homogenization of bacterial communities on local or regional levels. One of several examples that may support this hypothesis are seven closely related low-level *Pd*-inhibitors (AC1474_EST2874, AC1482_EST2887, AC162_JC353, AC1473_EST2873, AC161_JC352, AC208_CLC399, AC230_FW421) identified as strains of *Streptomyces corynorhini* [84] that were specific to ELMA, but were sampled across five different caves within the park and from two different bat species (*C*. *townsendii* and *M*. *thysanodes*, Fig. 3). Other work at ELMA sites exploring source-sink relationships of bat skin communities and their environment found that cave walls and cave air influence the bat microbiome and that the bat microbiome can have a reciprocal influence on cave walls [22]. Thus, bats may occasionally transport microbes to new environments, which could then pass on to other individuals or species. This phenomenon has been documented in other symbiont systems (e.g., bed bugs and bat flies) [85, 86].

A healthy skin microbiome can contribute to disease prevention both by occupying pathogen adhesion sites and producing pathogen inhibiting compounds [87]. While several researchers have investigated the impact of *Pd* presence on bat cutaneous microbial assemblage and structure, varied results across studies make identification of generalizable trends challenging; however, one consistent result is that *Pd* affects bacterial more than fungal communities [63, 88, 89]. Because certain taxa with antifungal capabilities appear to be enriched on *Pd* infected bats [63, 89, 90], it is anticipated that they may confer protection against WNS and reduce susceptibility of certain bats to the disease. These studies have yet to focus on members of *Actinomycetota* such as *Streptomyces*. Here, we add to previous research that confirm bat microbiomes possess members that have antifungal activity [1, 62, 66, 67, 91]. However, it remains unclear if certain taxa or combinations of taxa prevent severe disease, and future temporal studies evaluating changes of microbial community structure in relation to *Pd* loads and WNS disease progression may reveal the roles of bat-associated microbiota in pathogen defense. It is also likely that bat microbiomes vary between hibernation and active periods. However, investigating this is challenging, as disturbing bats during hibernation would be unethical and could cause unnecessary stress.

In addition to variations in microbiome assemblage and structure, mortality rates from WNS vary among bat species [92–94]. Bats across Eurasia have low *Pd* loads and infection prevalence, attributed in part to intrinsic and adaptive immune responses resulting from their coevolution with *Pd* over millennia [66, 95]. It is only in North America where *Pd* was introduced to native bat hosts that we see severe disease symptoms and death [96]. Even within the U.S., WNS disease susceptibility varies considerably. *Myotis* are among the most susceptible and impacted bats, particularly *M. lucifugus* [97] and *M. septentrionalis* [98, 99]. This is concerning for their western analogs *M. occultus* and *M. evotis*, respectively. We did not sample *M. occultus* in this study, but *M. evotis* had the fourth (out of 12) highest recovery rate of anti-*Pd* isolates. Rates of anti-*Pd* isolation among *Myotis* species varied greatly. *Myotis thysanodes* had the second highest anti-*Pd* rate while *M. californicus* had the second lowest (Fig. 5). Lower WNS impact rates are predicted for *M. velifer* [66, 100], *C. townsendii* [100, 101], *E. fuscus* [102, 103], *L. noctivagans* [5, 101, 104], but these projections are based on different criteria, and their power as predictors is variable. The vulnerability of *P. hesperus* may be similar to its eastern analog of *P. subflavus*, which is affected greatly by the disease [94, 105]. Here, we found *P. hesperus* had the third lowest (10th of 12) anti-*P. destructans* recovery rate. We found *T. brasiliensis* had the highest anti-*Pd* bacteria isolation rate of all bat species tested. *Tadarida brasiliensis* are shown to have same frequency of symptoms as highly impacted *M. lucifugus* under lab conditions, but with less severe pathology [106]. Because *T. brasiliensis* typically migrate south over the winter, it is anticipated that they will be less affected by WNS. While we identified bat species as an important factor in the recovery of *Pd* inhibiting bacteria from the bat skin microbiome (Table 2), we cannot say how this will affect vulnerability to WNS infections. At present, *M. velifer* appears to be most impacted by WNS in the Southwest experiencing declines in central Texas [107] and accounting for the highest number of reported deaths of any species in southeastern New Mexico [19]. However, it remains uncertain how these patterns may shift as an increasing number of species begin to show symptoms of WNS in the Southwest.

In addition to bat species, cave conditions will likely affect the success of *Pd* colonization on bat fur and skin, and, therefore, potential for mortality. Although New Mexico and Arizona have warm surface temperatures, the relatively stable subterranean temperature of cave hibernacula have appropriate conditions to sustain the fungus [28, 108, 109].

We acknowledge that there are several limitations of this study. In swabbing the skin and fur of bats, we were unable to conclude which members were functionally active versus transient spores. Culturing of bat-associated bacteria was biased towards those that can grow in laboratory conditions, and further, the use of selective media resulted in dominance of *Actinomycetota*. This approach was chosen to target species of *Streptomyces* known for their highly diverse and important natural products that generate some of the most widely utilized therapeutic pharmaceuticals [110, 111]. In addition to impacting the taxa isolated in our study, the choice of media also impacts the production of secondary metabolites by bacterial strains [112, 113]. As such, some species that were categorized as non-*Pd*-inhibitors may be capable of antifungal production under different culture conditions. Strains found to possess antifungal capabilities were only tested in co-culture against *Pd* rather than a complex host-associated community. Further research will need to be conducted to determine if the bioactivities identified in vitro translate to in vivo studies. With the recognition of at least two clades of *Pd* in Eurasia [114], it is important to note that these assays were performed with *Pd-1* clonal North American lineage, and that bioactivities may have different levels of effectiveness versus different *Pd *lineages. Several pure cultures were collected from each bat, and while we attempted to isolate only unique colonies, multiple strains with bioactivity from one bat could overestimate the presence of anti-*Pd* isolates. We could not identify taxa to species using solely the 16S rRNA gene [115], and species clustered at >99% sequence similarity may be several unique species [1]. Genomic studies promise both additional taxonomic and biosynthetic resolution. Organization of bat skin-associated microbes with bioactivity against *Pd* into a database, similar to efforts by scientists studying antifungal activity of amphibian-associated taxa to *Batrachochytrium dendrobatidis* [116], would be helpful to streamline future research objectives.

## Conclusions

The American southwest has a concentrated bat diversity that must be protected against ongoing threats of WNS. To our best knowledge, we provide the largest collection of members of the bat skin microbiome. This collection of nearly 3,000 isolates captured pre-WNS will provide baseline information before bat communities are impacted by the disease. This may include valuable insights into the impacts of the bat microbiome on disease resistance and severity, as well as enabling evaluation of changes to microbial community structure after arrival of WNS. The collection is dominated by the phylum *Actinomycetota* which contains several genera known for their production of bioactive natural products, and it will be a valuable resource for studies aimed at identifying novel natural products with diverse biological activities. Our results add to the growing evidence that the bat microbiome may play a role in protecting bats against WNS. Indeed, of the more than 1,500 bat-associated bacterial strains tested for antifungal activity, nearly 100 strains, primarily species of *Streptomyces*, were able to inhibit the growth of *Pd *in vitro*.* Prevalence of anti-*Pd* strains was significantly influenced by both ecoregion and bat species, highlighting regions and species hosting anti-*Pd* microbial communities that may mitigate WNS spread.

## Electronic supplementary material

Below is the link to the electronic supplementary material.

Supplementary material 1

Supplementary material 2

Supplementary material 3

Supplementary material 4

## Data Availability

The data generated from this study is available as a peer-reviewed data release [117].

## Abbreviations

WNS: White-nose syndrome
*Pd*: *Pseudogymnoascus destructans*
PARA: Grand Canyon-Parashant National Monument
SEAZ: Southeast Arizona Group of National Park Sites
ELMA: El Malpais National Monument
FS: Fort Stanton-Snowy River Cave National Conservation Area
BLM: Bureau of Land Management Sites
CAVE: Carlsbad Caverns National Monument
ANPA: *Antrozous pallidus*
COTO: *Corynorhinus townsendii*
EPFU: *Eptesicus fuscus*
LANO: *Lasionycteris noctivagans*
MYCA: *Myotis californicus*
MYCI: *Myotis ciliolabrum*
MYEV: *Myotis evotis*
MYTH: *Myotis thysanodes*
MYVE: *Myotis velifer*
MYVO: *Myotis volans*
PAHE: *Parastrellus hesperus*
TABR: *Tadarida brasiliensis*
